# Assignment of the Ile, Leu, Val, Met and Ala methyl group resonances of the DEAD-box RNA helicase DbpA from *E. coli*

**DOI:** 10.1007/s12104-020-09994-z

**Published:** 2020-12-04

**Authors:** Jan Philip Wurm

**Affiliations:** Department of Biophysics I, University of Regensburg, 93053 Regensburg, Germany

**Keywords:** DEAD-box helicase, Ribosome assembly, RNA, Methyl group assignment

## Abstract

ATP-dependent DEAD-box helicases constitute one of the largest families of RNA helicases and are important regulators of most RNA-dependent cellular processes. The functional core of these enzymes consists of two RecA-like domains. Changes in the interdomain orientation of these domains upon ATP and RNA binding result in the unwinding of double-stranded RNA. The DEAD-box helicase DbpA from *E. coli* is involved in ribosome maturation. It possesses a C-terminal RNA recognition motif (RRM) in addition to the canonical RecA-like domains. The RRM recruits DbpA to nascent ribosomes by binding to hairpin 92 of the 23S rRNA. To follow the conformational changes of Dbpa during the catalytic cycle we initiated solution state NMR studies. We use a divide and conquer approach to obtain an almost complete resonance assignment of the isoleucine, leucine, valine, methionine and alanine methyl group signals of full length DbpA (49 kDa). In addition, we also report the backbone resonance assignments of two fragments of DbpA that were used in the course of the methyl group assignment. These assignments are the first step towards a better understanding of the molecular mechanism behind the ATP-dependent RNA unwinding process catalyzed by DEAD-box helicases.

## Biological context

DEAD box helicases constitute the largest family of RNA helicases in eukaryotes and are found in all organisms (Fairman-Williams et al. 2010). They are key players in virtually every step of RNA biology and are implicated in infection and disease (Steimer and Klostermeier 2012).

**Fig. 1:**
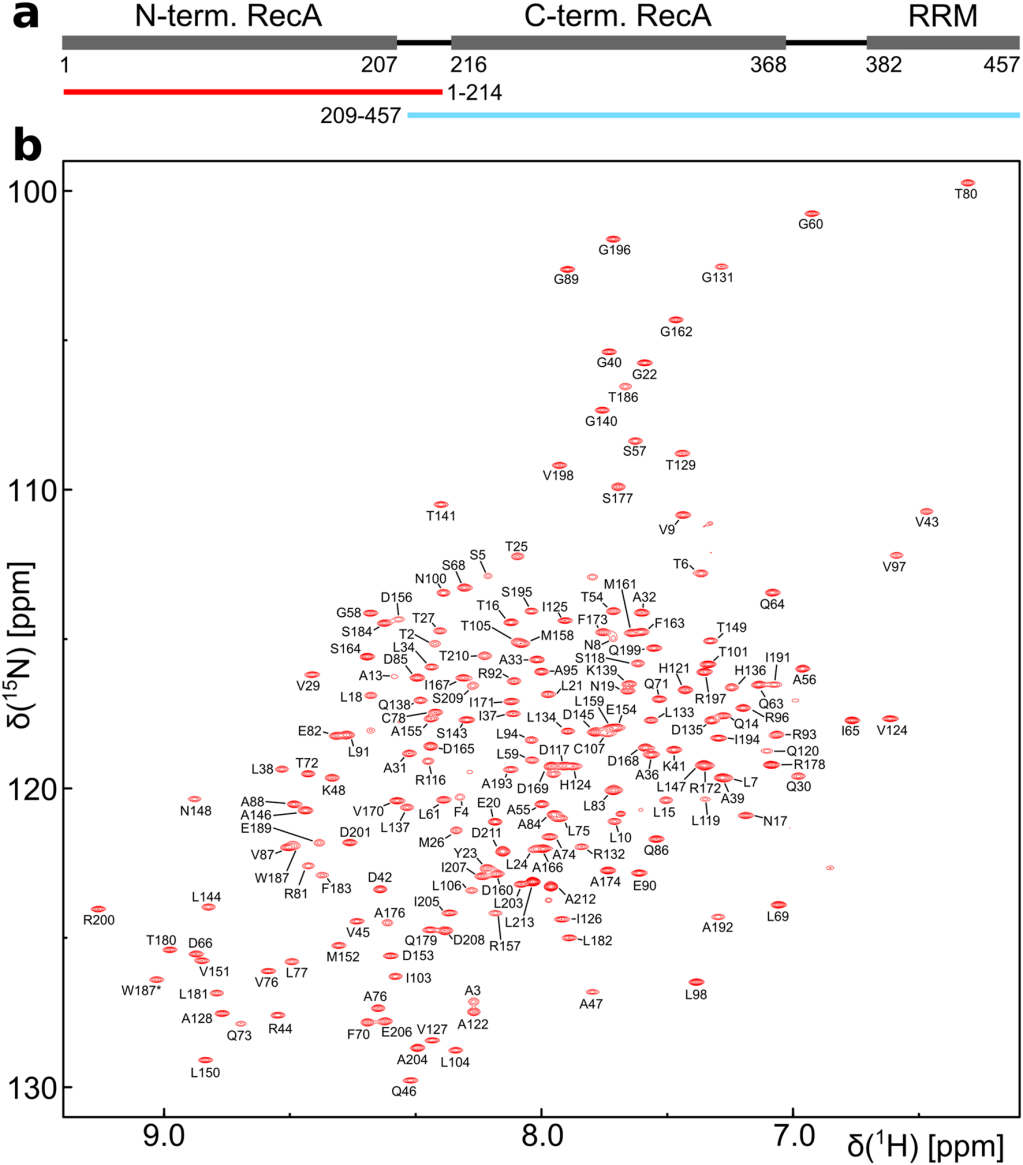
DbpA domain organization and backbone resonance assignment of the N-terminal construct of DbpA. **a** The positions of the RecA and RRM domains and their domain boundaries are shown. The N- (red) and C-terminal (blue) constructs that were used in the divide and conquer assignment approach are indicated below. **b** ^1^H^15^N-TROSY-HSQC spectrum of the ^15^N,^13^C labeled N-terminal construct (residues 1–214) recorded at 800 MHz. Assigned backbone amide signals are labeled. The side chain amide signal of Trp 187 is indicated by an asterisk

DEAD box helicases consist of two RecA-like domains (Fig. 1) and use the energy generated by ATP hydrolysis to unwind short stretches of duplex RNA (up to ~ 15–20 nt) in a nonprocessive manner (Fairman-Williams et al. 2010). The residues that are involved in ATP or RNA binding and the allosteric coupling between these two binding sites are highly conserved among all members of the enzyme family (Linder and Jankowsky 2011). The RNA unwinding activity is generally not sequence-specific, but DEAD box helicases usually possess flanking N- and C-terminal sequences that allow their selective recruitment to different cellular target sites (Fairman-Williams et al. 2010). Examples are sequence-specific RNA binding domains (Hardin et al. 2010) or short unstructured sequence motifs that are used to recruit helicases to their target site via protein–protein interactions (Sharma and Jankowsky 2014).

The unwinding mechanism of DEAD-box helicases has been extensively studied and is based on conformational changes between the RecA-like domains, which are connected by a flexible linker (Linder and Jankowsky 2011; Putnam and Jankowsky 2013). Based on single molecule FRET experiments (Theissen et al. 2008) and crystal structures of different DEAD-box helicases with wildly different interdomain orientations (e.g. (Caruthers et al. 2000; Story et al. 2001; Cheng et al. 2005)), it is generally assumed that the two RecA domains tumble independently in the apo state (Linder and Jankowsky 2011). Simultaneous binding of ATP and RNA induces the formation of a closed state, where ATP is buried between the two RecA domains and a bipartite RNA binding site is formed. This leads to the destabilization and thereby the unwinding of the RNA duplex (Putnam and Jankowsky 2013). In the post unwinding state (the helicase/ATP/single-stranded RNA complex) ATP is rapidly hydrolyzed to ADP and phosphate, which leads to the disassembly of the complex and allows for another round of unwinding (Theissen et al. 2008). Many structures of the apo and the post unwinding state (bound to single-stranded RNA and ATP-analogs) have been determined, but it is not clear how DEAD-box helicases initially interact with the double-stranded RNA substrate.

We recently initiated NMR studies of the *E. coli* DEAD-box helicase DbpA (UniProt.: P21693; 49.2 kDa; 457 residues). DbpA and its homolog YxiN from *B. subtilis* are model DEAD box helicases and have been used in numerous mechanistic and functional studies (Polach and Uhlenbeck 2002; Theissen et al. 2008; Henn et al. 2008, 2010; Aregger and Klostermeier 2009). DbpA is involved in ribosome biogenesis (Sharpe Elles et al. 2009) and possesses a C-terminal RNA recognition motif in addition to the canonical RecA domains (Hardin et al. 2010). The RRM specifically binds to hairpin 92 of the 23S rRNA and thereby recruits DbpA to the nascent ribosome (Tsu et al. 2001). Hairpin 92 is part of the highly conserved peptidyl transferase center which remains unstructured until the final stages of 50S ribosomal subunit maturation (Nikolay et al. 2018). The helicase activity of DbpA and YxiN is strongly activated upon binding of hairpin 92 to the RRM (Diges and Uhlenbeck 2001; Samatanga et al. 2017), but the molecular mechanism behind this allosteric activation process is not clear.

We report here the ILMVA methyl group assignment of full length DbpA. These assignments will serve as the basis for further studies on the mechanism behind the allosteric activation of DbpA and on the RNA unwinding mechanism. In this regard, it is noteworthy that neither the isolated C-terminal RecA domain nor the isolated RRM could be obtained in soluble form after expression in *E. coli*. A direct interaction between the RRM and the C-terminal RecA domain therefore seems likely and might be the basis for the allosteric activation of the helicase activity upon binding of hairpin 92 to the RRM.

## Methods and experiments

### Construct design

The gene coding for full length DbpA and two constructs comprising the N-terminal RecA domain (residues 1–214) or the C-terminal RecA domain plus the RRM (residues 209–457) were PCR amplified from genomic *E. coli* DNA (strain BL21(DE3)) and cloned into a modified pET vector with a TEV-cleavable, N-terminal hexahistidine tag. Point mutations were introduced using the QuickChange approach and verified by sequencing.

### Sample preparation

For protein expression the plasmids were transformed into *E. coli* BL21(DE3) cells. Cells were grown in M9 medium (H_2_O based for protonated samples or D_2_O based for deuterated samples) containing 0.5 g/l ^15^NH_4_Cl and 2 g/l glucose (^1^H^13^C-labeled for protonated samples, ^2^H^13^C-labeled for deuterated, uniformly ^13^C-labeled samples or ^2^H-labeled for deuterated samples with selective methyl group labelling). In order to adapt the cells to D_2_O-M9 medium the cells from an initial preculture in LB medium were transferred to 10% of the final volume of D_2_O-M9 medium to an OD_600_ of 0.15 and grown over night at 37 °C. This culture was used to inoculate the remaining D_2_O-M9 medium. Cells were grown to an OD_600_ of 0.7–0.9 at 37 °C, then IPTG was added to a concentration of 1 mM and proteins were expressed at 25 °C over night. Protonation of the methyl groups of Ile (Cδ methyl only), Val and Leu (ILV) in a ^2^H,^15^N,^13^C labeled background was achieved by addition of 60 mg/l 2-Ketobutyric acid-(^13^C_4_,3,3-d2) and 100 mg/l 2-Keto-3-methyl-butyric acid-(^13^C_5_, 3-d) to the medium. For ^1^H,^13^C labeling of the methyl groups of Ile (Cδ methyl only), Val, Leu, Met and Ala (ILMVA) in a ^2^H,^15^N labeled background 60 mg/l 2-Ketobutyric acid-(4-^13^C,3,3-d2), 100 mg/l 2-Keto-3-methyl-butyric acid-(dimethyl-^13^C_2_, 3-d), 100 mg/l L-methionine-(methyl-^13^C) and 100 mg/l L-alanine-(methyl-^13^C, 2-d) were added to the medium. All precursors were added 1 h prior to induction except for alanine, which was added 20 min before induction (Kerfah et al. 2015; Schütz and Sprangers 2020). Isotopically labeled precursors and amino acids were obtained from Cambridge Isotope Laboratories (Ile and Leu/Val precursors) or Sigma-Aldrich (L-methionine and L-alanine).

After expression cells were harvested by centrifugation, resuspended in buffer A (400 mM NaCl, 50 mM sodium phosphate, pH 7.4, 10 mM imidazole) supplemented with 0.1% (v/v) triton x-100 and 1 mg/ml lysozyme and lysed by sonication. Cell debris was removed by centrifugation and the supernatant was loaded onto a gravity flow Ni–NTA colum equilibrated in buffer A. The column was washed with wash buffer (1 M NaCl, 25 mM sodium phosphate, pH 7.4) to remove nucleic acids bound to DbpA and with buffer A supplemented with 20 mM imidazole. DbpA was eluted with elution buffer (150 mM NaCl, 25 mM sodium phosphate, pH 7.4, 300 mM imidazole). The hexahistidine tag was removed by TEV cleavage during dialysis over night at 4 °C against dialysis buffer (150 mM NaCl, 25 mM sodium phosphate, pH 7.4, 1 mM DTT). DbpA was loaded onto a NiNTA column equilibrated in dialysis buffer. The flow through was collected, mixed with ½ volume of 60% glycerol (v/v) and loaded onto a 5 ml HiTrap HP heparin column. DbpA was eluted using 10–50% gradient over 50 ml (buffer A: 25 mM HEPES, pH 7.3, 20% (v/v) glycerol, buffer B: as buffer A + 1 M NaCl). The heparin column was omitted for the N-terminal RecA domain as it lacks the positively charged RRM that is essential for binding to the heparin column. As the final purification step DbpA was subjected to size exclusion chromatography (SEC) using a Superdex 75 16/600 column (SEC buffer: 125 mM NaCl, 25 mM HEPES, pH 7.3, 1 mM DTT). NMR samples were prepared in SEC buffer supplemented with 5% (v/v) D_2_O.

### NMR experiments

The sample for the assignment of the N-terminal RecA domain (residues 1–214) was ^15^ N,^13^C labeled at a concentration of 400 μM. Higher concentrations lead to a strong decrease in signal intensity in the heteronuclear 3D spectra. The assignment of the N-terminal RecA domain was based on the following spectra: ^1^H^15^N-HSQC*, ^1^H^13^C-HSQC, 3D-HNCA, 3D-HN(CA)CO*, 3D-CBCA(CO)NH, 3D-HNCO*, 3D-HNCACB*, 3D-HBHA(CBCACO)NH, 3D-(H)CCH-TOCSY, 3D-H(C)CH-TOCSY, 3D-(H)CCH-COSY and 3D-H(C)CH-COSY (Sattler et al. 1999) (asterisks indicate TROSY based spectra (Salzmann et al. 1998)). The assignments from the ^15^ N,^13^C labeled sample were extended and transferred to the deuterated, ILMVA labeled sample based on a 3D-CCH-NOESY spectrum (SOFAST-HMQC-based, (Rossi et al. 2016)) and a model of the structure of the N-terminal RecA domain. The model was generated using the SWISS-MODEL server (Waterhouse et al. 2018) and the structure of the DEAD-box helicase VASA as template (pdb identifier 2DB3, (Sengoku et al. 2006)).

The sample for the assignment of the C-terminal construct (residues 209–457) was protonated at the methyl groups of Ile (Cδ methyl only), Val and Leu in a ^2^H,^15^N,^13^C labeled background at a concentration of 740 μM. To increase the solubility and long term stability of the sample the NaCl concentration was increased to 250 mM and 25 mM Arg/Glu was added to the sample buffer. The assignment of the backbone resonances and ILV methyl groups of the C-terminal construct was based on the following spectra: ^1^H^15^N-HSQC*, ^1^H^13^C-HMQC, 3D-HNCACB*, 3D-HN(CO)CACB*, 3D-HN(CA)CO*, 3D-HNCO*, 3D-(H)CC(CO)NH*, 3D-H(CCCO)NH*, 3D-HCH- and 3D-CCH-NOESY (SOFAST-HMQC-based, (Rossi et al. 2016)). The assignment was expanded to the Met and Ala methyl groups using a ILMVA ^1^H^13^C-methyl group labeled sample in a ^2^H,^15^N labeled background based on 3D-HCH-, 3D-CCH- and 3D-CNH-NOESY spectra in combination with models of the C-terminal RecA domain and the RRM. The models were generated based on the structures of the DbpA homolog YxiN from *Bacillus subtilis* (pdb identifiers 2HJV (Caruthers et al. 2006) and 3MOJ (Hardin et al. 2010) for the RecA and RRM domains, respectively).

The methyl group assignments of the two constructs could then be transferred to full length DbpA (residues 1–457) as the ^1^H^13^C-HMQC spectra of the individual domains overlay very well with the spectrum of the full length protein. Two methionine methyl group signals (M1 and M114) could not be assigned based on the recorded spectra. An M114E point mutation was thus introduced into full length DbpA. This allowed for the assignment of the M114 methyl group by comparison with the spectrum of wild type DbpA and also led to the assignment of the last unassigned methyl group signal as M1.

All spectra were recorded at 298 K on 600 and 800 MHz Bruker Neo Avance NMR spectrometers equipped with nitrogen (600 MHz) or helium cooled (800 MHz) cryoprobes. Spectra were processed with Topspin 4.0.2 and analyzed using CARA (Keller 2004).

## Assignments and data deposition

**Fig. 2:**
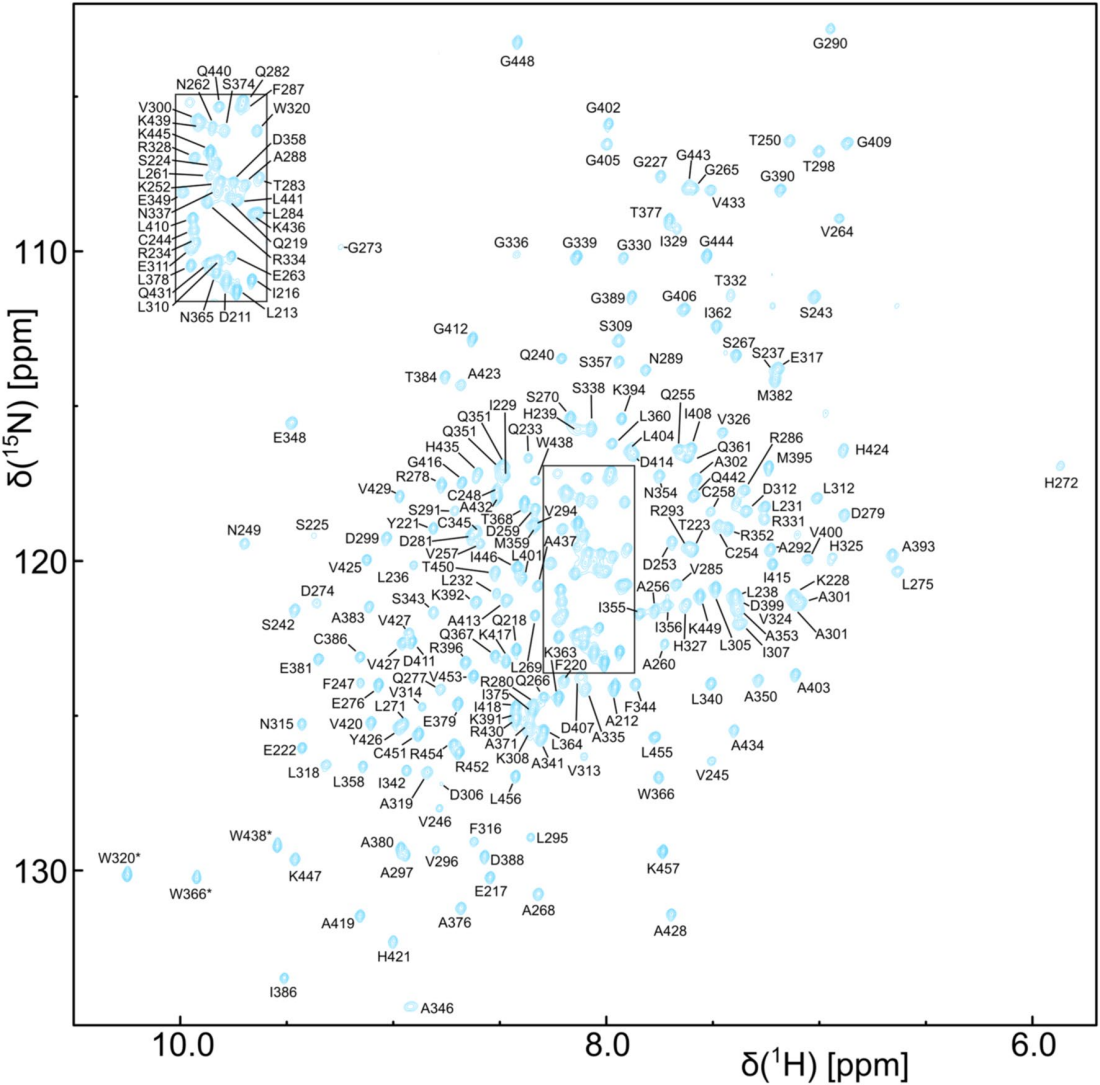
Backbone resonance assignment of the C-terminal construct of DbpA (residues 209–457) that comprises the C-terminal RecA domain and the RRM. ^1^H^15^N-TROSY-HSQC spectrum of a ^2^H,^15^N,^13^C labeled sample recorded at 800 MHz. Assigned backbone amide signals are labeled. Assigned Trp side chain amide signals are indicated by asterisks. The inset on the top left shows the boxed region in the center of the spectrum

**Fig. 3:**
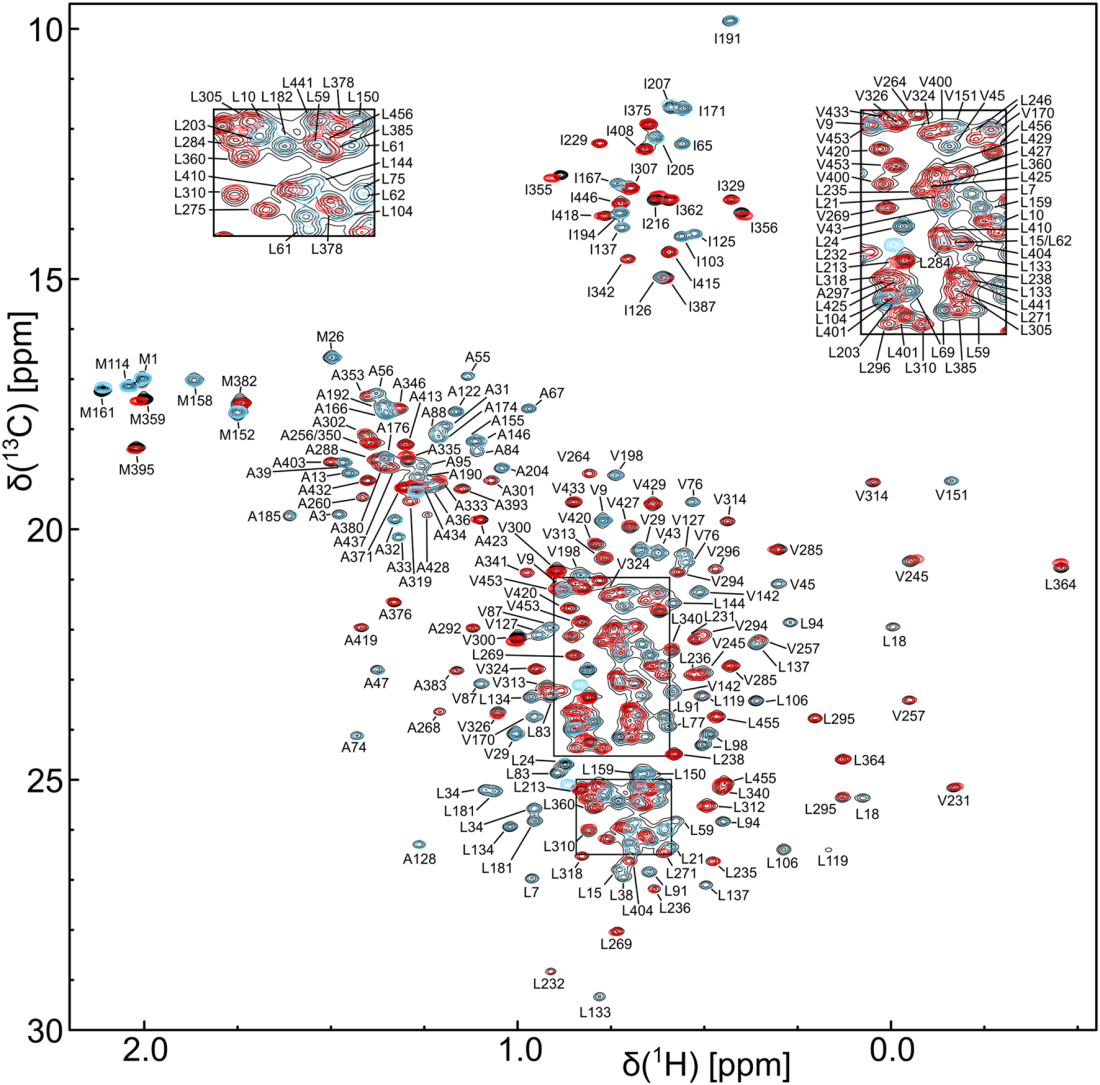
ILMVA methyl group assignments of DbpA. An overlay of the ^1^H^13^C-HMQC spectra of full length DbpA (black), the N-terminal construct (residues 1–214, blue) and the C-terminal construct (residues 209–257, red) is shown. The sum of the spectra of the isolated parts yields the spectrum of the full length protein. All spectra were recorded at 800 MHz on perdeuterated samples that are ^1^H,^13^C-labeleled at the ILMVA methyl groups. Assigned methyl group signals are labeled. The insets on the top show close-ups of the two boxed regions in the center of the spectrum

As the RecA domains of DEAD-box helicases have been shown to tumble independently in the apo state (Linder and Jankowsky 2011) we used a divide and conquer approach (Sprangers and Kay 2007) for the assignment (Fig. 1a). Two constructs of DbpA comprising the N-terminal RecA domain (residues 1–214) or the C-terminal RecA domain plus the RRM (residues 209–457) could be expressed and purified with high yield and showed well dispersed ^1^H^15^N TROSY-HSQC spectra (Figs. 1b, 2). In addition the ^1^H^13^C-HMQC spectra of the two constructs overlap very well the spectrum of full length DbpA (residues 1–457) (Fig. 3). This indicates that the RecA domains of the two constructs indeed do not interact with each other and allowed us to transfer the methyl groups assignments to full length DbpA.

For a ^15^N,^13^C labeled sample of the N-terminal RecA domain 88% of the H^N^, N, Cα, Cβ and C’ backbone resonances could be assigned (Fig. 1b). These assignments were extended to the side chain methyl groups using 3D-TOCSY and 3D-COSY experiments. Despite the relatively small size of the construct (22 kDa) HCC(CO)NH experiments yielded very low signal intensities. This is most likely the result of transient, nonspecific intermolecular interactions as increasing protein concentrations also lead to decreasing signal intensities in heteronuclear 3D spectra. The methyl group assignments obtained for the ^15^N,^13^C labeled sample were subsequently transferred to the perdeuterated, ILMVA labeled sample (Fig. 3) and extended to the Met methyl groups based on a 3D-CCH-NOESY experiment. In total 97% (134 out of 138) of the ILMVA methyl groups of the N-terminal RecA domain could be assigned.

For the assignment of the C-terminal construct (residues 209–457) a ^2^H,^15^N,^13^C labeled sample (protonated at the ILV methyl groups) was used that allowed for the assignment of 93% of the H^N^, N, Cα, Cβ and C’ backbone resonances (Fig. 2). Based on these assignments the majority of the ILV methyl groups were assigned using HCC(CO)NH type spectra. These assignments were then used as starting points for the assignment of the remaining ILMVA methyl groups (Fig. 3) based on 3D-NOESY spectra (HCH-, CCH- and CNH-type) and a homology model of the RecA and RRM domains.

As the sum of the methyl group spectra of the two assigned constructs is virtually identical to the spectrum of full length DbpA (Fig. 3) the methyl group assignments could directly be transferred to the full length protein. In total 98% (260 out of 266) of the ILMVA methyl groups of DbpA were assigned. The assignments have been deposited at the Biological Magnetic Resonance Bank under the accession numbers 50355 (N-terminal RecA domain), 50356 (C-terminal RecA domain plus RRM) and 50357 (full length DbpA).
